# Epidemiological Investigation of Tick-Borne Bacterial Pathogens in Domestic Animals from the Qinghai–Tibetan Plateau Area, China

**DOI:** 10.3390/pathogens13010086

**Published:** 2024-01-19

**Authors:** Yihong Ma, Yingna Jian, Geping Wang, Iqra Zafar, Xiuping Li, Guanghua Wang, Yong Hu, Naoaki Yokoyama, Liqing Ma, Xuenan Xuan

**Affiliations:** 1 National Research Center for Protozoan Diseases, Obihiro University of Agriculture and Veterinary Medicine, Obihiro 080-8555, Japan; 2 Qinghai Academy of Animal Sciences and Veterinary Medicine, Centre for Biomedicine and Infectious Diseases, Qinghai University, Xining 810016, China; 3 Veterinary Research Institute, Livestock and Dairy Development Department, Lahore 54810, Pakistan

**Keywords:** tick-borne bacterial pathogens, livestock, Qinghai–Tibetan Plateau area

## Abstract

The Qinghai–Tibetan Plateau area (QTPA) features a unique environment that has witnessed the selective breeding of diverse breeds of domestic livestock exhibiting remarkable adaptability. Nevertheless, *Anaplasma* spp., *Rickettsia* spp., *Coxiella* spp., and *Borrelia* spp. represent tick-borne bacterial pathogens that pose a global threat and have substantial impacts on both human and animal health, as well as on the economy of animal husbandry within the Qinghai–Tibetan plateau area. In this study, a total of 428 samples were systematically collected from 20 distinct areas within the Qinghai Plateau. The samples included 62 ticks and 366 blood samples obtained from diverse animal species to detect the presence of *Anaplasma* spp., *Rickettsia* spp., *Coxiella* spp., and *Borrelia* spp. The prevalence of infection in this study was determined as follows: *Anaplasma bovis* accounted for 16.4% (70/428), *A. capra* for 4.7% (20/428), *A. ovis* for 5.8% (25/428), *Borrelia burgdorferi* sensu lato for 6.3% (27/428), *Coxiella burnetii* for 0.7% (3/428), and *Rickettsia* spp. for 0.5% (2/428). Notably, no cases of *A. marginale* and *A. phagocytophilum* infections were observed in this study. The findings revealed an elevated presence of these pathogens in Tibetan sheep and goats, with no infections detected in yaks, Bactrian camels, donkeys, and horses. To the best of our knowledge, this study represents the first investigation of tick-borne bacterial pathogens infecting goats, cattle, horses, and donkeys within the Qinghai Plateau of the Qinghai–Tibetan Plateau area. Consequently, our findings contribute valuable insights into the distribution and genetic diversity of *Anaplasma* spp., *Rickettsia* spp., *Coxiella* spp., and *Borrelia* spp. within China.

## 1. Introduction

The Qinghai–Tibetan Plateau area (QTPA), boasting the highest average elevation globally, harbors a diverse range of ungulate species, comprising approximately 42% of the total ungulate species in China [1]. The significant variations in altitude and the diverse climatic conditions of the plateau have fostered a multitude of ecological environments, which, in turn, have created ecological niches for a wide range of high-altitude species, including different kinds of domestic animals [2]. Nonetheless, the sensitivity of the Qinghai–Tibetan Plateau to the effects of global climate change has been noted [3].

The QTPA is home to a diverse range of livestock species that have adapted to survive in high altitude and cold climate conditions [4]. Among these unique species are Tibetan sheep (*Ovis aries*), goats (*Capra hircus*), yaks (*Bos grunniens*), cattle (*Bos taurus domestica*), horses (*Equus ferus caballus*), camels (*Camelus bactrianus*), and donkeys (*Equus asinus*) [5]. Within the QTPA, particular livestock species coexist alongside thriving ticks that exhibit adaptability to various environmental niches, characterized by their sensitivity to temperature and humidity conditions [6]. Ticks serve as primary vectors for various pathogens worldwide, impacting both wildlife and domestic animals. While the majority of these pathogens are specific to animals, certain tick-borne diseases are zoonotic, posing significant health risks to humans and, in some cases, leading to severe or fatal consequences [7]. Previous studies have identified the presence of 2 families, 6 genera, and 31 distinct species of ticks in the Qinghai Plateau of the QTPA. Notably, *Haemaphysalis qinghaiensis* and *Dermacentor nuttalli* are the predominant tick species found in Qinghai province and are widespread across most regions [8]. Consequently, the presence of tick-borne bacterial pathogens, including *Anaplasma*, *Borrelia*, *Rickettsia*, and *Coxiella,* poses a significant concern in this region.

Anaplasmosis is caused by a group of important tick-borne bacteria in the genus *Anaplasma*. In China, anaplasmosis is generally found in livestock. *A. platys* was detected in Bactrian camels in China [9]. Furthermore, the occurrence of *A. bovis* in sheep, goats and yaks, *A. ovis* in sheep and goats, *A. phagocytophilum* in yaks, as well as *A. marginale* in cattle, has been reported within China [10,11,12,13]. Notably, *A. bovis* and *A. ovis* can cause anaplasmosis in cattle and sheep, with a high infection rate among livestock; the infection rate of sheep in Haixi and Haibei has reached 58% [14].

Lyme disease is a global natural zoonotic disease caused by *Borrelia burgdorferi* [15]. In China, at least 14 species of *B. burgdorferi* sensu lato have been identified in ticks, animals, and humans, providing evidence of the wide distribution and diversity of *B. burgdorferi* s.l. across China [16]. Results of an epidemiological survey conducted in the forested region of Qinghai Plateau revealed a higher prevalence of Lyme disease in the agricultural area as compared to the pastoral area [17].

An earlier study has indicated the presence of *B. burgdorferi* s.l. in tick hosts, including humans, yaks, cattle, mice, and Tibetan sheep [18].

*Rickettsia* spp., categorized under the spotted fever group (SFG), have been identified as causative agents of zoonotic diseases affecting humans, domestic animals, and wildlife, thus emerging as a significant global health concern [19]. In China, several species, including *R. heilongjiangensis*, *R. sibirica* subsp. sibirica BJ-90, *R. raoultii*, *R. japonica*, *Rickettsia* sp. XY99, and *Candidatus Rickettsia tarasevichiae*, have been reported [20,21,22,23,24,25,26]. While *Rickettsia* is predominantly present in ticks within the Qinghai Plateau [19,27,28,29], there is limited information regarding its incidence in domestic animals.

*Coxiella burnetii*, the causative agent of Q fever, is a significant zoonotic pathogen with a wide distribution [30]. Infection of *C. burnetii* in goats has been identified as a major reservoir for human infection. Furthermore, infections in cattle have been associated with cases of abortion and have shown higher prevalence rates in older animals [31]. A study conducted in the Qinghai Plateau region reported the detection of *C. burnetii* within ticks [14].

Previous research has primarily focused on the detection of pathogens in ticks. However, limited information is available regarding the incidence of these pathogens in domestic animals, particularly goats, cattle, horses, and donkeys in the QTPA. In order to gain a comprehensive understanding of bacterial microorganisms associated with ticks in various domestic animals in the Qinghai–Tibetan Plateau area, China, we performed targeted molecular screening to identify the presence of bacterial pathogens. Therefore, the current study aims to investigate the incidence of tick-borne bacterial pathogens that infect both ticks and seven distinct types of livestock within the Qinghai–Tibetan Plateau area, providing a comprehensive understanding of the prevalence and distribution of pathogens within the study area.

## 2. Materials and Methods

### 2.1. Blood Sample Collection

**Figure 1:**
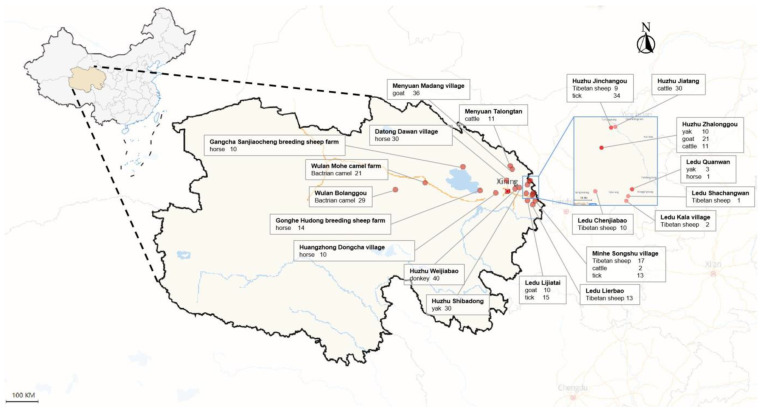
A map of the Qinghai Plateau highlighting the various sampling sites and animals included. The figure was created and adjusted using map data in Excel.

A comprehensive collection of 366 randomly obtained blood samples was assembled, representing a diverse range of livestock species, including 52 Tibetan sheep (*Ovis aries*), 67 goats (*Capra hircus*), 43 yaks (*Bos grunniens*), 49 cattle (*Bos taurus domestica*), 40 donkeys (*Equus ferus caballus*), 50 camels (*Camelus bactrianus*), and 65 horses (*Equus asinus*). These samples were gathered from 20 distinct locations situated within the Qinghai Plateau of QTPA (Figure 1). Prior to including livestock in this study, the farm owner was asked for their willingness to participate in the research activity and verbal consent was obtained.

### 2.2. Morphological and Molecular Identification of Ticks

A total of 62 adult tick samples were collected from three distinct locations within the Qinghai Plateau of the Qinghai–Tibetan Plateau area (QTPA), specifically Jinchangou in Huzhu County, Lijiatai Village in Ledu County, and Songshu Village in Minhe County (Figure 1). Tick samples were directly collected from domestic animals’ external surfaces and obtained using the Flagging method, involving systematic cloth sweeping across vegetation in the field. All tick specimens were carefully preserved in sampling vials and promptly transported to the laboratory for detailed analyses. The identification of each tick specimen was conducted based on morphological criteria, following the methodology outlined by Chen et al. [32]. Additionally, the identification was further confirmed through sequence analyses of a partial fragment of the cytochrome c oxidase subunit I (*coxI*) gene. A fragment of approximately 850 base pairs (bp) within the *coxI* gene was amplified using polymerase chain reaction (PCR) using the primers *coxI* F (5′-GGAACAATATATTTAATTTTTGG-3′) and *coxI* R (5′-ATCTATCCCTACTGTAAATATATG-3′) [33].

### 2.3. DNA Extraction

The TIANamp Genomic DNA Kit (TIANGEN, Beijing, China) was employed in strict adherence to the manufacturer’s guidelines. For tick samples, the midgut and salivary glands were separated, while for blood samples, whole blood with an anticoagulant (EDTA) was processed using the same extraction protocol. The concentration of DNA was determined using a Biochrom WPA Biowave DNA Life Science Spectrophotometer (Biochrom, Cambridge, UK), and the DNA samples were subsequently stored at −20 °C until their future utilization.

### 2.4. Molecular Identification of Tick-Borne Bacterial Pathogens

Tick-borne bacterial pathogens were detected and characterized by PCR in Tibetan sheep, goats, yaks, cattle, donkeys, horses, Bactrian camels, and ticks from the 20 sites of the Qinghai Plateau using the primers listed in Table 1. The PCR reaction was performed with a total volume of 10 μL, containing 1 μL of the DNA template, 0.5 μL each of the forward and reverse primers (100 μM), 0.2 μL of deoxyribonucleotide triphosphate (200 μM; New England BioLab, Ipswich, Massachusetts, USA), 1 μL of 10× ThermoPol Reaction Buffer (New England BioLab, Ipswich, Massachusetts, USA), 0.1 μL of Taq polymerase (0.5 U; New England BioLab, Ipswich, Massachusetts, USA), and double-distilled water up to 10 μL [34]. A negative control was included, using double-distilled water instead of DNA template. The PCR products were visualized by UV transillumination in a 1.5% agarose gel following electrophoresis and staining with ethidium bromide.

### 2.5. Sequencing Analyses of Detected Tick-Borne Bacterial Pathogens

For the detection of tick-borne bacterial pathogens, at least one positive sample per animal species within each sampling area was selected for sequencing and subsequent molecular characterization. The PCR product obtained from the positive sample was purified using the EasyPure Quick Gel Extraction Kit (TransGen Biotech, Beijing, China) and subsequently cloned into *E. coli* DH5α via the pMD™18-T Vector Cloning Kit (Takara, shiga, Japan). At a minimum, three positive clones were sent for sequencing services provided by Genewiz (Suzhou, China) company.

### 2.6. Phylogenetic Tree of Detected Tick-Borne Bacterial Pathogens

The acquired sequences were validated through a BLASTn search in GenBank (https://blast.ncbi.nlm.nih.gov/Blast.cgi (accessed on 9 June 2023)). Subsequently, phylogenetic trees were constructed using the Maximum Likelihood statistical method and 1000 replications of bootstrap analyses through MEGA X: Molecular Evolutionary Genetics Analysis version X, designed for larger datasets [45].

### 2.7. Statistical Analyses

The proportion of samples tested positive between different animals was compared by Epitools online (https://epitools.ausvet.com.au (accessed on 15 August 2023)). The chi-squared test for the contingency table was applied to the original data to quantify the prevalence of each pathogen’s infection rate, along with a corresponding 95% confidence interval (CI). Observed differences were considered statistically significant when the resulting *p*-values were lower than 0.05.

## 3. Results

### 3.1. Morphological and Molecular Identification of Ticks

**Figure 2:**
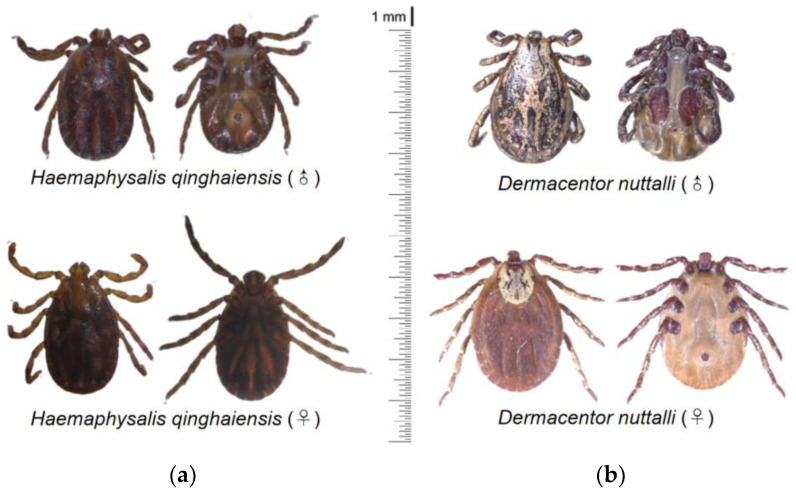
Morphological identification of ticks in QTPA. (**a**) *H. qinghaiensis* detected in QTPA; (**b**) *D. nuttalli* from QTPA in this study.

**Figure 3:**
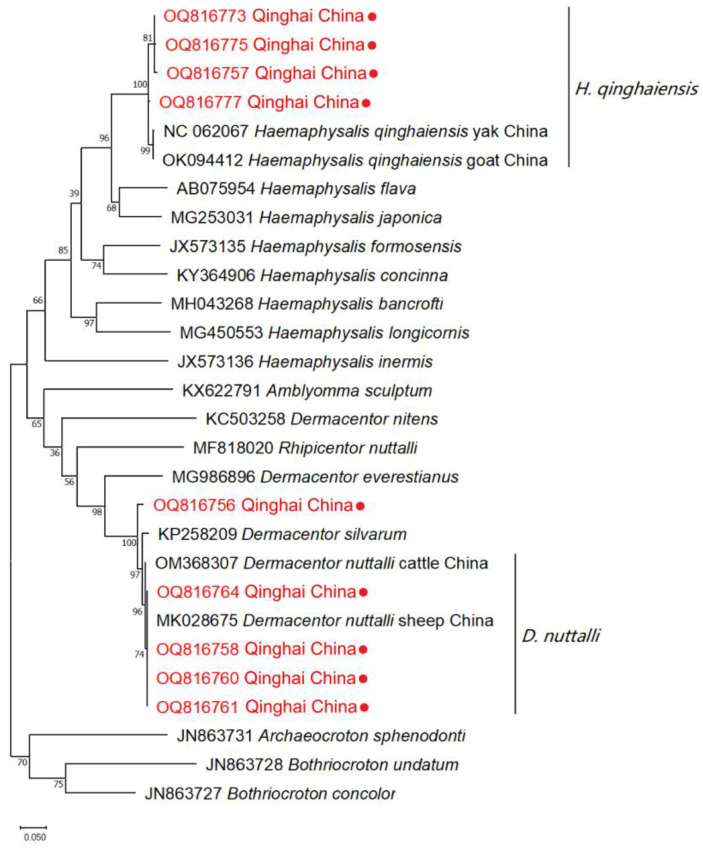
Phylogenetic tree of ticks using the Maximum Likelihood method. The numbers indicated at the nodes represent the percentage of occurrence of clades based on 1000 bootstrap replications of the data. The sequences of isolates obtained in this study, along with their corresponding accession numbers, are highlighted in red.

A light microscope was utilized for the observation of tick species under 40 × magnification. The results of the morphological identification of ticks revealed the detection mainly of two primary species within the Qinghai Plateau, namely *H. qinghaiensis* and *D. nuttalli* (Figure 2). A total of 23 ticks were submitted to the Genebank. After conducting rigorous sequence alignment analyses, our investigation has primarily provided evidence of the presence of two distinct tick species, *H. qinghaiensis* and *D. nuttalli*, in the Qinghai Plateau region, with nucleotide consistency levels ranging from 97.53–100% (Table 2). The results of the phylogenetic analyses indicate that based on the *coxI* gene, the tick species obtained in this study can be mainly classified into two clades: one clade is closely related to *H. qinghaiensis*, while the other is closely related to *D. nuttalli* from China (Figure 3). In this investigation, we have observed that OQ816756 exhibits notable dissimilarity when compared to the *D. nuttalli* and *D. silvarum* clade. It is suggested that OQ816756 could potentially represent novel variants within the *Dermacentor* genus.

### 3.2. Identification of Tick-Borne Bacterial Pathogens

*A. bovis*, *A. capra*, *A. marginale*, *A. ovis*, *A. phagocytophilum*, *B. burgdorferi* s.l., *C. burnetii*, and *Rickettsia* spp. transmitted by ticks were investigated. As presented in Table 3, the prevalence of infection was found to be 16.4% (70/428) for *A. bovis*, 4.7% (20/428) for *A. capra*, 5.8% (25/428) for *A. ovis*, 6.3% (27/428) for *B. burgdorferi* s.l., 0.7% (3/428) for *C. burnetii*, and 0.5% (2/428) for *Rickettsia* spp. No cases of infections caused by *A. marginale* and *A. phagocytophilum* were detected. Additionally, no instances of tick-borne bacterial pathogen infections were found in yaks, Bactrian camels, donkeys, and horses among the livestock examined. The prevalence of tick-borne bacterial pathogens in the dominant animals showed no statistically significant difference (chi-squared test, *p* > 0.05).

### 3.3. Sequencing and Comparative Analyses

The sequences obtained in the present investigation were analyzed using the Basic Local Alignment Search Tool (BLAST) at the National Center for Biotechnology Information (NCBI). Thirteen sequences of *Anaplasma bovis*, two of *A. ovis*, eight of *A. capra*, two of *Rickettsia* spp., two of *B. burgdorferi* s.l., and two sequences of *C. burnetii* were submitted to the Genebank. The results presented in this study include 24 distinctive sequences of tick-borne bacterial pathogens (Table 4).

The investigation into tick-borne bacterial pathogens has revealed a high degree of sequence similarity among the identified species of *Anaplasma*, ranging from 99.27% to 100% for *A. bovis*, 99.71% to 100% for *A. ovis*, and 97.58% to 99.62% for *A. capra*. For the identified species of *Rickettsia* spp. (*R. raoultii*), the sequence similarity was 100%. The identified species of *Borrelia* (KY284014) exhibited sequence similarity ranging from 99.52% to 99.84%, and the identified species of *C. burnetii* (MK416231) showed a sequence similarity of 99.69%.

### 3.4. Phylogenetic Analyses

**Figure 4:**
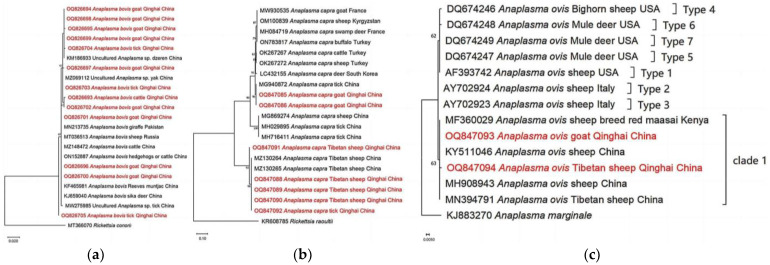
Phylogenetic trees of *Anaplasma* spp. constructed using the Maximum Likelihood method in MEGA X, employing the Kimura 2-parameter model. (**a**) The phylogenetic tree of *A. bovis* based on 16s rRNA gene. (**b**) The phylogenetic tree of *A. capra* based on *gltA* genes. (**c**) The phylogenetic of *A. ovis* based on *msp4* gene. The numbers assigned to the nodes represent the percentage of occurrence of clades, determined through 1000 bootstrap replications of the data. Isolates from this study, along with their corresponding accession numbers, are highlighted in red.

Based on our analyses of the 16S rRNA gene, the isolates of *A. bovis* obtained from goats, cattle, and ticks exhibited a close genetic relationship with previously reported *A. bovis* isolates. However, it is noteworthy that the isolate OQ826705 displayed distinct dissimilarity compared to *A. bovis*, suggesting its potential classification as a novel variant within the species *A. bovis* (Figure 4a). Additionally, our phylogenetic analyses based on partial sequences of the citrate synthase gene (*gltA*) revealed interesting findings. They showed that *A. capra* isolates obtained from goats in our study are genetically related to *A. capra* isolates previously detected from ticks in China [46]. Moreover, the analyses demonstrated that *A. capra* isolates derived from Tibetan sheep and ticks are positioned within the same clade as Tibetan sheep isolates obtained from Qinghai, as reported by He et al. [47]. These results suggest a genetic similarity and potential association between *A. capra* variants found in Tibetan sheep, ticks, and the Tibetan sheep population from the Qinghai region (Figure 4b). Moreover, our phylogenetic analyses of *A. ovis*, based on the Major surface protein 4 (*msp4)* partial sequences obtained from Tibetan sheep and goats, revealed the presence of a new clade, distinct from those previously identified in a study conducted by de la Fuente et al. [48] (Figure 4c).

**Figure 5:**
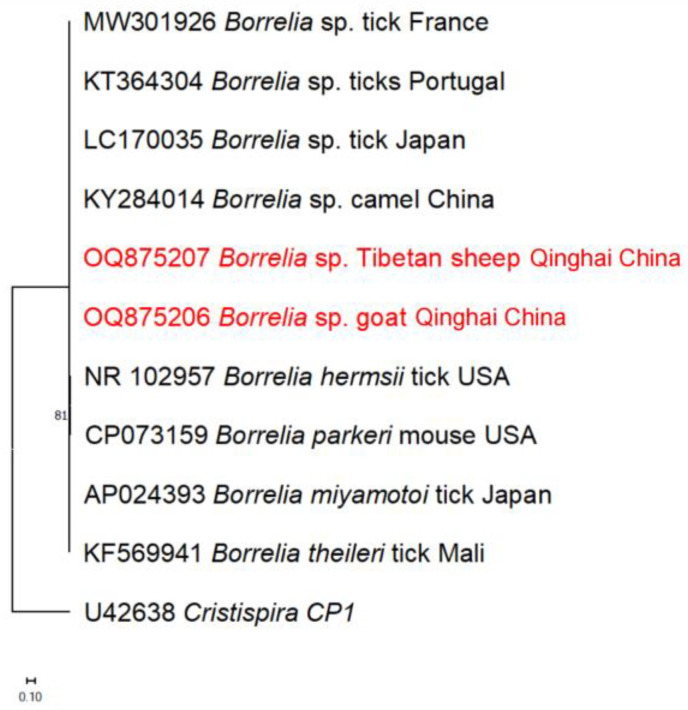
The phylogenetic tree of *B. burgdorferi* s.l., based on 16s rRNA partial sequences obtained from Tibetan sheep and goats in this study, as well as sequences retrieved from the GenBank database, was constructed using the Maximum Likelihood method in MEGA X and Kimura 2-parameter model. The numbers assigned to the nodes indicate the percentage of occurrence of clades, determined through 1000 bootstrap replications of the data. Isolates from this study, along with their corresponding accession numbers, are highlighted in red.

The 16S rRNA sequences of *B. burgdorferi* s.l. obtained from Tibetan sheep and goats in this study were found to cluster with homologous sequences from camels in China (Figure 5).

**Figure 6:**
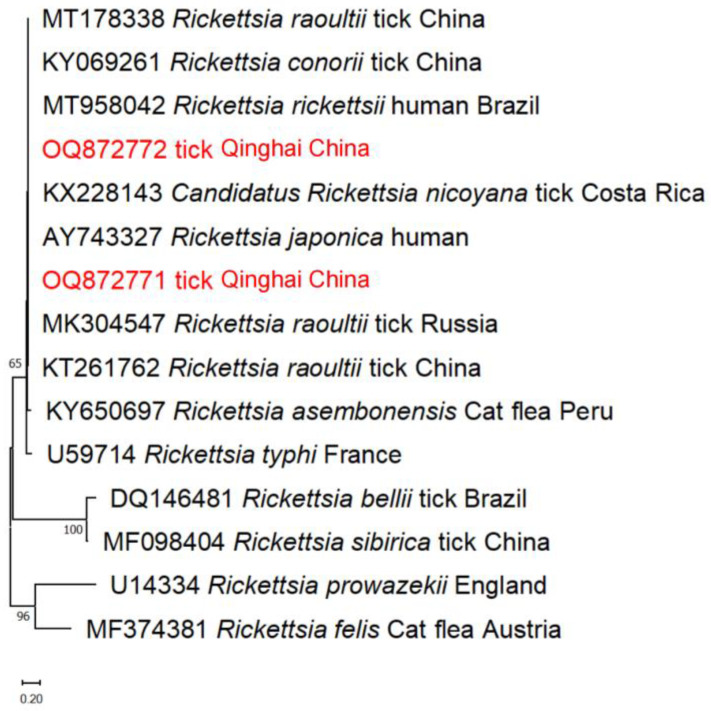
The phylogenetic tree of *Rickettsia* spp., constructed using the Maximum Likelihood method in MEGA X and employing the Tamura 3-parameter model, includes numbers assigned to the nodes representing the percentage of occurrence of clades and was determined through 1000 bootstrap replications of the data. Isolates from this study, along with their corresponding accession numbers, are highlighted in red.

Furthermore, the phylogenetic analyses of *Rickettsia* spp. based on the *gltA* gene revealed that the tick isolates obtained in this study belong to the *R. raoultii* clade, along with ticks from China and Russia (Figure 6).

**Figure 7:**
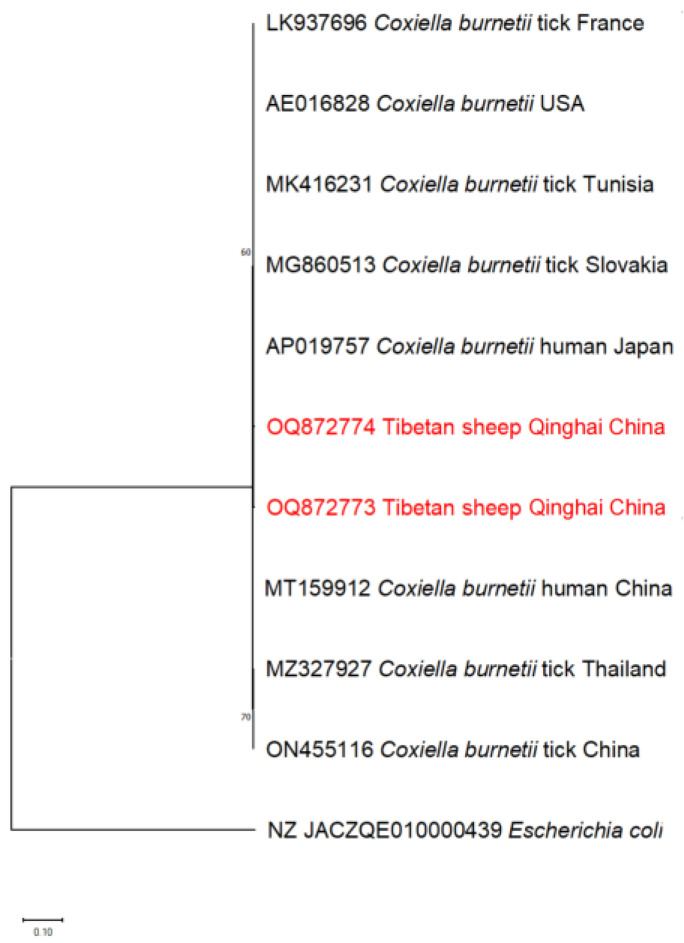
The phylogenetic tree of *C. burnetii*, constructed using the Maximum Likelihood method in MEGA X and employing the Kimura 2-parameter model, includes numbers assigned to the nodes representing the percentage of occurrence of clades and was determined through 1000 bootstrap replications of the data. Isolates from this study, along with their corresponding accession numbers, are highlighted in red.

Moreover, the *C. burnetii* based on heat shock protein B (*htpB*) was found to be in the same clade as those from ticks and humans in other countries (Figure 7). The detection of similar clade membership across diverse geographic locations underscores the potential for transnational transmission.

## 4. Discussion

The Qinghai–Tibetan Plateau region is characterized by vast grasslands, towering mountains, and serene lakes, constituting a unique geographical environment. The landscape of this region is intricately intertwined with diverse livestock species that rely on the grasslands for grazing, establishing a symbiotic relationship with the natural surroundings. Moreover, ticks, known to inhabit grasslands, forests, and shrubbery, are also prevalent in this ecosystem. Ticks play a crucial role as carriers of human and animal pathogens globally. Notable examples of these tick-borne bacterial pathogens, with implications for both veterinary and public health, include *Rickettsia* spp., *Anaplasma phagocytophilum*, *Borrelia burgdorferi* sensu lato (s.l.), and *Coxiella burnetii*. These pathogens have the capacity to infect a diverse array of hosts, encompassing humans as well as wild and domestic animals [49]. While various studies have investigated the prevalence of tick-borne pathogens in different regions [50,51,52,53], limited research has been conducted that specifically focuses on the Qinghai–Tibetan Plateau [14,18,19]. To date, this study represents the first comprehensive investigation into the presence of tick-borne bacterial pathogens in this unique geographical region.

The investigation of tick-borne diseases may depend significantly on the distribution and diversity of tick species. Individuals bitten by *Haemaphysalis* ticks carrying bacterial infections face increased risk of bacterial infection [53]. Some tick species have successfully adapted to the dry and cold climate of the plateau, such as *H. qinghaiensis* [54], which is the predominant tick species in Qinghai province, China. Our study confirmed the presence of *H. qinghaiensis* and *D. nuttalli*, aligning with previous research findings [8]. To date, there has not been a comprehensive and organized investigation, leading to a lack of knowledge about various pathogens in Qinghai province. This study employed molecular detection methods to investigate the prevalence of these pathogens among major livestock species in Qinghai province. The primary objective of this study was to ascertain the presence of tick-borne pathogens and acquire a more profound comprehension of their biological characteristics and classification within the province. Furthermore, it facilitates research into the biological traits of potential novel tick-borne pathogens.

In the context of *Anaplasma* spp., this investigation has provided valuable insights into the geographical distribution of diverse tick-borne bacterial pathogens in China. Importantly, it marks the first identification of *A. bovis*, *A. capra*, and *A. ovis* in goats, as well as the detection of *A. bovis* in cattle within the Qinghai Plateau. Notably, Tibetan sheep and goats displayed a high susceptibility to *Anaplasma* infections, with a remarkable 65.7% of goats found to be infected with *A. bovis*. Among the five different *Anaplasma* species tested, the highest infection rate was observed for *A. bovis*, with 16.4% of the samples testing positive. The presence of *A. phagocytophilum* has been previously documented in Gansu province [55], while *A. marginale* has been detected in both Sichuan province [56] and the Tibet Autonomous Region [57], which shares a border with the Qinghai Plateau. Additionally, ticks from Qinghai have also tested positive for both *A. marginale* and *A. phagocytophilum* [46,58]. However, contrary to these findings, the present study observed the absence of these pathogens.

Based on the results of this study, only two instances of *Rickettsia* spp. infection were detected in the ticks. Detailed analyses of the genetic sequences unveiled 100% similarity between the positive sequences and the previously identified *R. raoultii* isolates originating from Russia and China. It is worth noting that earlier studies had already established the presence of *R. raoultii* in ticks from the Qinghai province [19,27,28], further confirming our findings.

The findings regarding *C. burnetii* reveal that Tibetan sheep and ticks in Qinghai demonstrate a seropositivity rate of 0.5% (2/428). It is noteworthy that the sequencing analyses revealed a remarkable 99.69% similarity to the tick sample obtained from Tunisia (MK416231). This finding differs from the strain of *C. burnetii* previously detected in ticks from the Qilian area of Qinghai province [13]. Moreover, phylogenetic tree analyses further establish that these organisms belong to the same clade as those found in foreign nations, strongly suggesting the possibility of pathogen importation from other countries. The identification of *C. burnetii* in neighboring provinces [59,60,61] and the Qinghai Plateau implies the widespread presence of this bacterium in diverse geographical regions, indicating its potential for transmission to both animals and humans across a broader geographic scope.

In this study, the presence of *B. burgdorferi* s.l. was detected in Tibetan sheep and goats. It is worth mentioning that while previous studies have reported various animal species being infected with *B. burgdorferi* s.l. [18], this study represents the first documented instance of its detection in goats within the Qinghai region.

In summary, this study represents the inaugural exploration of tick-borne pathogens in goats, horses, and donkeys within the QTPA. It offers crucial insights into the prevalence and genetic diversity of *Anaplasma* spp., *Rickettsia* spp., *Coxiella* spp., and *Borrelia* spp. throughout China. The significance of these pathogens lies in their zoonotic potential, indicating the risk of transmission from animals to humans. The intricate interplay between animal and human ecosystems underscores the imperative for robust surveillance and prevention strategies to alleviate the impact of these zoonotic tick-borne bacteria on health and productivity. This study reveals the zoonotic risk of tick-borne pathogens in the Qinghai–Tibetan Plateau, emphasizing the need for public health awareness, veterinary vigilance, and conservation efforts. The findings underscore international health security concerns and advocate for a comprehensive One Health approach, urging further research and collaborative strategies. Nevertheless, it is essential to acknowledge certain limitations inherent in this study, including its focused scope, potential diagnostic biases, and the absence of long-term monitoring capabilities, which may impact its generalizability or applicability beyond specific contexts or regions. Consequently, future research initiatives should conscientiously address these constraints to foster a more comprehensive understanding of tick-borne infections, not only within the QTPA but potentially extending to broader geographical contexts as well.

## Figures and Tables

**Table 1:** The list contains the sequences of the PCR primers used in tick-borne bacterial pathogens.

Species	Target Gene	Method	Primer Sequence		Annealing Temperature (°C)	Amplicon Size (bp)	Reference
*A. bovis*	16S rRNA	PCR	F	TCCTGGCTCAGAACGAACGCTGGCGGC	55	1433	[35]
			R	AGTCACTGACCCAACCTTAAATGGCTG			
		nPCR ^1^	nF ^2^	CTCGTAGCTTGCTATGAGAAC	55	551	[36]
			nR ^3^	TCTCCCGGACTCCAGTCTG			
*A. capra*	*gltA*	PCR	F	GCGATTTTAGAGTGYGGAGATTG	55	1031	[9]
			R	TACAATACCGGAGTAAAAGTCAA			
		nPCR	nF	GGGTTCMTGTCYACTGCTGCGTG	55	793	
			nR	TTGGATCGTARTTCTTGTAGACC			
*A. marginale*	*msp4*	PCR	F	CTGAAGGGGGAGTAATGGG	60	344	[37]
			R	GGTAATAGCTGCCAGAGATTCC			
*A. ovis*	*msp4*	PCR	F	TGAAGGGAGCGGGGTCATGGG	62	347	[37]
			R	GAGTAATTGCAGCCAGGCACTCT			
*A. phagocytophilum*	16S rRNA	PCR	F	CACATGCAAGTCGAACGGATTATTC	55	932	[38]
			R	TTCCGTTAAGAAGGATCTAATCTCC			
		nPCR	nF	AACGGATTATTCTTTATAGCTTGCT	55	546/565	
			nR	GGCAGTATTAAAAGCAGCTCCAGG			
*B. burgdorferi* s.l.	23S rRNA	PCR	F	GCGAACGGGTGAGTAACG	50	1360	[39]
			R	CCTCCCTTACGGGTTAGAA			
	16S rRNA	PCR	F	GAGGCGAAGGCGAACTTCTG	60.2	622	[40]
			R	CTAGCGATTCCAACTTCATGAAG			
*C. burnetii*	*htpB*	PCR	F	GCGGGTGATGGTACCACAACA	57	501	[41]
			R	GGCAATCACCAATAAGGGCCG			
		nPCR	nF	TTGCTGGAATGAACCCCA	52	325	
			nR	TCAAGCTCCGCACTCATG			
*Rickettsia* spp.	*ompB*	PCR	F	AAACAATAATCAAGGTACTGT	55	212/209	[42]
			R	TACTTCCGGTTACAGCAAAGT			
	*ompA*	nPCR	nF	GCTTTATTCACCACCTCAAC		811	[43]
			nR	TR(g/a)ATCACCACCGTAAGTAAAT			
	*gltA*	nPCR	nF	GCAAGTATCGGTGAGGATGTAAT		401	[44]
			nR	GCTTCCTTAAAATTCAATAAATCAGGAT			

^1^ nPCR = nested polymerase chain reaction. ^2^ nF = nested forward primer. ^3^ nR = nested reverse primer.

**Table 2:** Accession numbers assigned to the isolates of ticks in the Qinghai Plateau of QTPA.

Tick Species	Target Gene	GenBank Accession Number	Length (bp)	Identity (%)	Accession Number (Host, Country)
*D. nuttalli*	*coxI*	OQ816756	849	97.76%	OM368307 cattle China
*H. qinghaiensis*	*coxI*	OQ816757	849	97.76%	NC_062067 yak China
*D. nuttalli*	*coxI*	OQ816758	849	100%	MK028675 sheep China
*H. qinghaiensis*	*coxI*	OQ816759	849	99.18%	NC_062067 yak China
*D. nuttalli*	*coxI*	OQ816760	849	100%	MK028675 sheep China
*D. nuttalli*	*coxI*	OQ816761	849	100%	MK028675 sheep China
*D. nuttalli*	*coxI*	OQ816762	849	100%	MK028675 sheep China
*D. nuttalli*	*coxI*	OQ816763	849	100%	MK028675 sheep China
*D. nuttalli*	*coxI*	OQ816764	849	99.88%	MK028675 sheep China
*D. nuttalli*	*coxI*	OQ816765	849	100%	MK028675 sheep China
*D. nuttalli*	*coxI*	OQ816766	849	100%	MK028675 sheep China
*D. nuttalli*	*coxI*	OQ816767	849	100%	MK028675 sheep China
*D. nuttalli*	*coxI*	OQ816768	849	100%	MK028675 sheep China
*D. nuttalli*	*coxI*	OQ816769	849	100%	MK028675 sheep China
*D. nuttalli*	*coxI*	OQ816770	849	100%	MK028675 sheep China
*D. nuttalli*	*coxI*	OQ816771	849	99.88%	MK028675 sheep China
*D. nuttalli*	*coxI*	OQ816772	849	100%	MK028675 sheep China
*H. qinghaiensis*	*coxI*	OQ816773	849	98.12%	OK094412 goat China
*D. nuttalli*	*coxI*	OQ816774	849	98.59%	OM368307 cattle China
*H. qinghaiensis*	*coxI*	OQ816775	849	98.23%	OK094412 goat China
*H. qinghaiensis*	*coxI*	OQ816776	849	98.12%	OK094412 goat China
*H. qinghaiensis*	*coxI*	OQ816777	849	98.82%	OK094412 goat China
*D. nuttalli*	*coxI*	OQ816778	849	97.53%	OM368307 cattle China

**Table 3:** The prevalence of tick-borne bacterial pathogens in livestock from Qinghai Plateau.

TBPs ^1^	Sheep	goat	Cattle	Yak	Camel	Donkey	Horse	Tick	*p*-Value
	52	67	49	43	50	40	65	62	
*A. bovis*	n. d. ^2^	44 (65.67; 12.02) ^3^	1 (2.04; 0.27)	n. d.	n. d.	n. d.	n. d.	3 (4.83; 0.82)	0.29
*A. capra*	8 (15.38; 2.19)	10 (14.93; 2.73)	n. d.	n. d.	n. d.	n. d.	n. d.	2 (3)	0.29
*A. marginale*	n. d.	n. d.	n. d.	n. d.	n. d.	n. d.	n. d.	n. d.	-
*A. ovis*	24 (46.15; 6.56)	1 (1.49; 0.27)	n. d.	n. d.	n. d.	n. d.	n. d.	n. d.	0.31
*A. phagocytophilum*	n. d.	n. d.	n. d.	n. d.	n. d.	n. d.	n. d.	n. d.	-
*B. burgdorferi* s.l.	14 (26.92; 3.83)	13 (19.40; 3.55)	n. d.	n. d.	n. d.	n. d.	n. d.	n. d.	0.31
*C. burnetii*	2 (3.84; 0.55)	n. d.	n. d.	n. d.	n. d.	n. d.	n. d.	1 (1.61; 0.27)	0.31
*Rickettsia* spp.	n. d.	n. d.	n. d.	n. d.	n. d.	n. d.	n. d.	2 (3.23; 0.55)	0.33

^1^ TBPs = tick-borne pathogens. ^2^ n.d. = not detected. ^3^ Number of positives (positives/specific livestock %; positives/total livestock %).

**Table 4:** Accession numbers for sequences of tick-borne bacterial pathogens deposited in GenBank.

Obtained Sequences					The Closest BLASTn Match		
Pathogen	Animal	Target Gene	GenBank Accession Number	Length (bp)	Identity (%)	Pathogen Isolate	Accession Number (Host, Country)
*A. bovis*	cattle	16s rRNA	OQ826693	551	99.64%	Uncultured *Anaplasma* sp.	KM186933 dzeren China
	goat	16s rRNA	OQ826694	551	99.82%	Uncultured *Anaplasma* sp.	KM186933 dzeren China
	goat	16s rRNA	OQ826695	551	99.64%	Uncultured *Anaplasma* sp.	KM186933 dzeren China
	goat	16s rRNA	OQ826696	548	100%	*A. bovis*	MN213735 giraffe Pakistan
	goat	16s rRNA	OQ826697	551	99.82%	Uncultured *Anaplasma* sp.	MZ069112 yak China
	goat	16s rRNA	OQ826698	551	99.82%	Uncultured *Anaplasma* sp.	KM186933 dzeren China
	goat	16s rRNA	OQ826699	551	99.82%	Uncultured *Anaplasma* sp.	KM186933 dzeren China
	goat	16s rRNA	OQ826700	551	100%	*A. bovis*	MN213735 giraffe Pakistan
	goat	16s rRNA	OQ826701	551	100%	*A. bovis*	MN213735 giraffe Pakistan
	goat	16s rRNA	OQ826702	551	99.82%	Uncultured *Anaplasma* sp.	MZ069112 yak China
	tick	16s rRNA	OQ826703	551	100%	Uncultured *Anaplasma* sp.	MZ069112 yak China
	tick	16s rRNA	OQ826704	551	99.27%	Uncultured *Anaplasma* sp.	KM186933 dzeren China
	tick	16s rRNA	OQ826705	551	99.46%	*A. bovis*	MN213735 giraffe Pakistan
*A. ovis*	goat	msp4	OQ847093	347	100%	*A. ovis*	MN394791 Tibetan sheep China
	Tibetan sheep	msp4	OQ847094	347	99.71%	*A. ovis*	MN394791 Tibetan sheep China
*A. capra*	tick	gltA	OQ847092	793	99.37%	*A. capra*	MZ130266 Tibetan sheep China
	goat	gltA	OQ847085	793	98.63%	*A. capra*	MW930535 goat France
	goat	gltA	OQ847086	793	98.49%	*A. capra*	MW930535 goat France
	Tibetan sheep	gltA	OQ847088	793	99.75%	*A. capra*	MZ130266 Tibetan sheep China
	Tibetan sheep	gltA	OQ847089	793	99.62%	*A. capra*	MZ130266 Tibetan sheep China
	Tibetan sheep	gltA	OQ847090	793	99.62%	*A. capra*	MZ130264 Tibetan sheep China
	Tibetan sheep	gltA	OQ847091	793	97.86%	*A. capra*	MZ130266 Tibetan sheep China
*Rickettsia* spp.	tick	gltA	OQ872771	400	100%	*R. raoultii*	MK304547 tick Russia
	tick	gltA	OQ872772	400	100%	*R. raoultii*	MT178338 tick China
*Borrelia* spp.	goat	16s rRNA	OQ875206	623	99.52%	*Borrelia* sp.	KY284014 camel China
	Tibetan sheep	16s rRNA	OQ875207	623	99.84%	*Borrelia* sp.	KY284014 camel China
*C. burnetii*	tick	htpB	OQ872773	325	99.69%	*C. burnetii*	MK416231 tick Tunisia
	Tibetan sheep	htpB	OQ872774	325	99.69%	*C. burnetii*	MK416231 tick Tunisia

## Data Availability

All data are disclosed in the paper.
